# Authors' Reply

**DOI:** 10.1371/journal.pmed.0030067

**Published:** 2006-01-31

**Authors:** Bertran Auvert, Joëlle Sobngwi-Tambekou, Dirk Taljaard, Emmanuel Lagarde, Adrian Puren

**Affiliations:** **1**Hôpital Ambroise-Paré, Assistance Publique—Hôpitaux de Paris, Boulogne, France; INSERM U 687, Saint-Maurice, France; University Versailles Saint-Quentin, Versailles, France; IFR 69, Villejuif, France; **2**INSERMSaint-MauriceFrance; **3**ProgressusJohannesburgSouth Africa; **4**Rémi Sitta, IFR 69, Villejuif, France and INSERM U 687, Saint-Maurice, France; **5**National Institute for Communicable DiseaseJohannesburgSouth Africa

Jennifer Vines raises the question of other potential sources of HIV [ 1]. During our follow-up, 569 participants received blood transfusions, were hospitalized, or received injections. We observed 15 infections among those who had such a nosocomial risk factor during the period when HIV infection occurred. We observed 50 infections among those without such a risk factor. In a multivariate analysis of risk factors of HIV infection during the follow-up, the presence of a nosocomial risk factor was not significantly associated with HIV infection (rate ratio [RR] = 1.7; *p* = 0.092). Among those with a nosocomial risk factor, the protective effect of the intervention (intention-to-treat analysis) is about 58%. Among those without a nosocomial risk factor, the protective effect of the intervention is about 62%. In a multivariate analysis, when taking into account the nosocomial risk factors, the association between group of randomization and HIV infection was unchanged (protection of 60% versus 60%). The fact that patients with a nosocomial risk factor were not significantly more at risk of HIV infection, and were protected by male circumcision in a similar way to those without a nosocomial risk factor, strongly supports the view that the majority of HIV infections observed in our study were due to sexual transmission.

John Potterat and colleagues make a number of points to which we must respond [ 2]. The association between clinic attendance for a health problem related to genitals and HIV infection is most likely due to genital herpes, which is common in South Africa and strongly associated with HIV. We believe that those who became HIV positive also became infected with HSV-2 just before, at the same time, or just after the acute primary HIV infection. Primary genital herpes infection concomitant with HIV infection can lead to clinic attendance because of herpes genital lesions, and can explain the observed association.

In this population of young men, as shown in Table 4 of our Research Article [ 3], we did not observe any significant association between reported sexual behaviour characteristics and HIV infection when controlling for other factors, including the randomisation group. However, in univariate analysis, there is an association between HIV infection and number of sexual contacts (RR = 2.0; *p* = 0.035) and risk of infection (RR = 1.7; *p* = 0.045). This last variable includes lack of condom use. We believe that because of the small number of infections, and the importance of male circumcision on the transmission of HIV, the factors associated with sexual behaviour do not appear in the multivariate analysis. In addition, we think that the HIV status of female partners of these young men is a key factor. In Table 4, it is clear that HIV infection increases with the age of the participants, which is a proxy for the risk of having a partner who is infected with HIV for two reasons: (1) because the age of female partners increase with age of their male partners and (2) because, in young women, HIV status is strongly associated with age.

We did ask the sex of all reported sexual partners. We found that 0.07% of these reported partners were men. Three participants reported sexual partnerships with men. None became infected during the follow-up.

At recruitment, HIV prevalence was 0.7% (two out of 278) among those who reported never having any sexual contact, and 4.8% (144 out of 2,994) among those who reported having sexual contact ( *p* < 0.001).

HIV incidence was about two out of 100 per year in the control group during the follow-up. Knowing that HIV incidence increases with age, and assuming that the incidence is negligible before 17, we can estimate the HIV incidence between the age of 17 (median age of first sexual experience) and the age of 21 (age at recruitment) by about half the incidence of the control group during the follow-up, leading to an estimated HIV incidence of one out of 100 per year. It means that the HIV prevalence of the participants between this four-year period (17–21) goes up from 0% to 4%. We observed about 4.5% at recruitment, which is consistent with this analysis.

We agree with the comment by James Shelton [ 4]. When looking at the crossovers, we found that they were protected by male circumcision. This implies that the difference between the intention-to-treat analysis and the per-protocol analysis is at least partly caused by the dilution effect of crossovers. Therefore, we believe, as does Shelton, that the 60% degree of protection obtained from the intent-to-treat analysis is probably too conservative.

We also agree with the points made by Adamson S. Muula and would like to note that, for many researchers, the results of this trial are not surprising and are consistent with many other studies published since 1986 [ 5].

With reference to the comment by Hugh Young [ 6], we reported analyses in our paper to account for this six-week period and for differences of behaviour between the two arms of the trial. These analyses show that the six-week period of abstinence cannot explain the outcome of the trial, and that participants in the intervention group were slightly more sexually active. Therefore, we strongly believe that the difference in rate of infection can be attributed to male circumcision. We think the study design suggested by Young would be unlikely to obtain ethical approval and would be unacceptable to participants.

Michael Glass has read a number of reports of our study. During the trial, we collected about 12,000 blood samples, performed about 12,000 clinical examinations, and collected about 48,000 questionnaires. We were careful to enter all these data with a double-entry procedure and even a triple-entry procedure for the laboratory data. This, of course, took considerable time. Nevertheless, we wanted to make available to the international community some preliminary information as soon as possible. We decided to release the results of the trial in a preliminary form at the International AIDS Society Conference in Rio de Janeiro. It is often the case that the results presented in a conference do not correspond exactly with those presented in the abstract, and that the final published results can be slightly different from those given in the oral presentation. We knew that this might be a problem, and we were careful to indicate to the *PLoS Medicine* editors that the results would be finalized only after the conference.

In addition, there is not just one numerical value but two: the result given by the per-protocol analysis and the result given by the intention-to-treat analysis, each having a wide confidence interval.

The magnitude of the effect in a trial of this nature cannot, in any case, predict precisely what to expect in an actual intervention program for four reasons. Firstly, the trial was conducted in a specific population (young men of the age range 18–24); it was not representative of the whole population. Secondly, the participants received intense counselling periodically throughout the follow-up period. Thirdly, they were informed that the result of the trial was unpredictable. Finally, the duration of the follow-up period was short. This is why operational research should be conducted to test if male circumcision, in association with existing and validated prevention methods, can be used in a community intervention.

Richard Winkel has also raised a number of concerns [ 7]. Our participants were recruited among the general population of the area. This neither implies that they are representative of this population nor that they are very different. HIV prevalence among those who were recruited is similar to what was expected, and clinical examination rarely revealed a tight foreskin. We do agree that the results obtained in this trial have to be confirmed by other trials, but also, as mentioned above, by conducting operational research.

Winkel seems to believe that the foreskin has only recently been linked to the transmission of infectious diseases. Hutchinson in 1854 noticed an association between male circumcision and a lower rate of syphilis. The first paper on the association between male circumcision and HIV infection was published in 1986. In addition, it is possible that male circumcision was practiced by the Egyptians, for health reasons, at least 3,000 years ago. Contrary to what Winkel has written, researchers are working on the effect of male circumcision on male-to-female transmission, despite technical difficulties. The ongoing trial in Uganda should yield new knowledge on this issue.

The aim is not to conduct a “new medical crusade against normal human anatomy” [ 7]. It is to reduce the mortality due to AIDS by reducing the spread of HIV, especially in the worst-infected countries.

In response to Jonathan Sykes [ 8], we would like to point out that several well-conducted meta-analyses and systematic reviews have shown that male circumcision is associated with lower rates of HIV infection among males. These studies have been quoted in our paper reporting our trial. It is rarely argued that male circumcision might worsen the epidemic, even if the safety of male circumcision is a real problem. The main remaining scientific problems are (1) to have the result of the South African study confirmed by the other ongoing trials and (2) to demonstrate that safe male circumcision is protective at population level by conducting applied research studies.

We can assure Mpho Selemogo [ 9] that we did consider the issue he raises very seriously when designing the trial. The guidelines of the HREC (the ethics committee of the University of the Witwatersrand) are quite clear that participation should be voluntary and that there should be no evidence of inducement. The minimum basic salary in South Africa is roughly R1,500 per month, which translates to about R70 per day. The R300 that we gave participants was distributed in increments across five visits. For the inclusion visit, participants received R30. For the three-month visit, they received R40; for the 12-month visit, R80; and for the final visit, R150. On average, participants received R75 per visit. The primary reasoning for an incremental compensation was because of the duration of the trial, and also to balance requirements of cohort retention. However, the increments were within the ethical bounds set by the HREC. In addition, we thought that it would eliminate the risk of recruiting people wishing to participate for immediate financial gain. We asked all participants why they wanted to be part of the study. A very small percentage (0.3%) indicated that they were participating for financial reward. The majority participated for the safe and free circumcision and to improve their health, 37.7% and 40%, respectively.

The honoraria paid during this trial compare very favourably to those paid to participants in other trials in the region. (For a drug trial, the current compensation, as stipulated by the Medicines Control Council, is R150 per visit.)

Finally, we do agree with Taiwo Lawoyin [ 10]. More research is needed to fill the gaps in this area. Nevertheless, we hope that these gaps will not be used as arguments to delay the use of male circumcision in slowing the spread of HIV in those countries of sub-Saharan Africa that have high rates of HIV infection and low rates of male circumcision, and where acceptability studies have already revealed a potential for this prevention method. Male circumcision should always be used in combination with other validated prevention methods.
